# A strategy for designing voriconazole dosage regimens to prevent invasive pulmonary aspergillosis based on a cellular pharmacokinetics/pharmacodynamics model

**DOI:** 10.1186/s12967-018-1533-4

**Published:** 2018-06-07

**Authors:** Taotao Wang, Tao Zhang, Ti Meng, Ying Li, Lu Chen, Qianting Yang, Haiyan Dong, Jin’e Lei, Limei Chen, Yalin Dong

**Affiliations:** 1 Department of Pharmacy, The First Affiliated Hospital of Xi’an Jiaotong University, Xi’an, 710061 China; 2 Department of Laboratory, The First Affiliated Hospital of Xi’an Jiaotong University, Xi’an, 710061 China; 3 Department of Hematology, The First Affiliated Hospital of Xi’an Jiaotong University, Xi’an, 710061 China

**Keywords:** Voriconazole, Invasive pulmonary aspergillosis, Prophylactic antifungal regimen, Monte Carlo simulation, Cellular pharmacodynamic/pharmacodynamic

## Abstract

**Background:**

Invasive pulmonary aspergillosis (IPA) is a life-threatening disease in immunosuppressed patients. Voriconazole is commonly used to prevent and treat IPA in the clinic, but the optimal prophylactic antifungal regimen is unknown. The objective of this study was to clarify the mechanism underlying how voriconazole prevents IPA based on a target cellular pharmacokinetics/pharmacodynamics model, with the aim of identifying a way to design an optimal prophylactic antifungal regimen.

**Methods:**

A nystatin assay was used to establish a target-cells model for *A. fumigatus* infection. An inhibitory effect sigmoid E_max_ model was developed to explore the cellular PK/PD breakpoint, and Monte Carlo simulation was used to design the prophylactic antifungal regimen.

**Results:**

The intracellular activity of voriconazole in the target cells varied with its concentration, with the minimum inhibitory concentration (MIC) being an important determinant. For *A. fumigatus* strains AF293 and AF26, voriconazole decreased the intracellular inoculum by 0.79 and 0.84 lg cfu, respectively. The inhibitory effect sigmoid E_max_ model showed that 84.01% of the intracellular inoculum was suppressed by voriconazole within 24 h, and that a PK/PD value of 35.53 for the extracellular voriconazole concentration divided by MIC was associated with a 50% suppression of intracellular *A. fumigatus*. The Monte Carlo simulation results showed that the oral administration of at least 200 mg of voriconazole twice daily was yielded estimated the cumulative fraction of response value of 91.48%. Concentration of voriconazole in the pulmonary epithelial lining fluid and the plasma of > 17.77 and > 1.55 mg/L, respectively, would ensure the PK/PD > 35.53 for voriconazole against most isolates of *A. fumigatus* and may will be benefit to prevent IPA in clinical applications.

**Conclusions:**

This study used a target cellular pharmacokinetics/pharmacodynamics model to reveal a potential mechanism underlying how voriconazole prevents IPA and has provided a method for designing voriconazole prophylactic antifungal regimen in immunosuppressed patients.

## Background

Invasive pulmonary aspergillosis (IPA) is mainly caused by *A. fumigatus* and has a high morbidity and mortality in high-risk immunosuppressed patients [1]. The incidence of IPA varies according to the underlying disease, being up to 24% in patients with acute leukemia, up to 10% in patients with allogeneic hematopoietic stem cell transplantation, and up to 7% in patients with lymphoid malignancies [2]. Additionally, the mortality rate of IPA is reportedly as high as 36–75% in patients with hematological malignancies despite the application of antifungal agents such as triazoles and echinocandins [3–5]. Thus, antifungal prophylaxis is currently recommended in high-risk immunosuppressed patients [6, 7].

Currently, no reported study has investigated how to develop an optimal antifungal prophylactic dosage regimen, and so the most common practice in clinical is to apply the recommended dosage regimen for prophylaxis. Theoretically, an understanding of the pathogenesis of *A.* *fumigatus* could provide clues for designing prophylactic antifungal regimen that are effective at preventing IPA. Inhalation is the primary route for acquiring *Aspergillus* spores. Pulmonary epithelial cells are the first cells encountered by the inhaled spores, which then germinate and grow in these cells, followed by the development of serious IPA disease in immunosuppressed patients [8]. Therefore, pulmonary epithelial cells can be viewed as the target of prophylaxis in IPA.

Voriconazole is a triazole exhibiting broad-spectrum antifungal activity against *Aspergillus* species [9], and is approved as the first-line therapy for invasive aspergillosis [6, 10]. The Infectious Diseases Society of America recommends using voriconazole for prophylaxis against invasive aspergillosis in high-risk patients, such as patients with prolonged neutropenia, patients with graft-versus-host disease, and lung transplant patients (strong recommendation and moderate-quality evidence) [6]. In addition, voriconazole is safe and effective for the secondary prophylaxis of systemic fungal infection in patients receiving allogeneic stem cell transplantation [11]. It has been reported that the concentration of voriconazole in the pulmonary epithelial lining fluid (ELF) crucially influences the prevention of IPA [12]. However, the ability of voriconazole penetrate the ELF varies widely in patients, with concentrations ranging from 0 to 83 mg/L being found in transplant patients receiving the recommended oral dosage regimen [13, 14]. While the ELF is on the surface of the pulmonary epithelial cells [15], the activity of voriconazole against *A.* *fumigatus* in these cells is unclear, which hinders the ability to design an optimal prophylactic dosage regimen for voriconazole in immunosuppressed patients.

We established a model of infected human pulmonary epithelial cells caused by *A.* *fumigatus* conidia with the following aims: (1) to determine the cellular pharmacokinetics/pharmacodynamics (PK/PD) characteristics of voriconazole in pulmonary epithelial cells, and (2) to identify a strategy for designing prophylactic regimen of voriconazole that are effective at preventing IPA.

## Methods

### Cell line, strains, and drug preparation

Human pulmonary epithelial cells (A549) were obtained from Shanghai Institute of Cell Biology in the Chinese Academy of Sciences (Shanghai, China). A549 cells were grown in vitro using 1640 medium supplemented with 10% fetal bovine serum and 1% penicillin/streptomycin, respectively. *A. fumigatus* strain AF293 (from FGSC, Fungal Genetics Stock Center, School of Biological Sciences, University of Missouri, Kansas City, Missouri, USA) and clinical isolate strain (AF26, identified by department of laboratory, The First Affiliated Hospital of Xi’an Jiaotong University) were used for our study and grown on Sabouraud Dextrose Agar (SDA) medium at 35 °C for 5 days. For these two strains, conidia were harvested using phosphate-buffered saline plus 0.1% Tween 80. Voriconazole and nystatin were purchased from the national institute for Food and Drug control (Beijing, China) and dissolved in dimethyl sulfoxide.

### Antifungal susceptibility testing

The *E*-test was performed according to the manufacturer’s instructions. The *E*-test strips (Biomeriux, USA) contained voriconazole at concentrations ranging from 0.002 to 32 mg/L. *E*-test strips were placed on SDA and the plates were incubated at 35 °C, and the minimum inhibitory concentration (MIC) was determined after an incubation time of 48 h. The MIC determined by the *E*-test was the lowest drug concentrations at which the border of the elliptical inhibition intercepted the scale on the antifungal strip.

### Cellular PK/PD properties of voriconazole

#### Infected model of A549 cells

A549 cells were seeded at 5 × 10^5^ cells/well in six-well plates and grown for 24 h. Following cell growth and washing by PBS, A549 cells and conidia were combined at a 1:5 ratio in six-well plates and incubated for 3 h at 37 °C. After incubation, the cells were washed three times with PBS and then treated with nystatin-supplemented media for a further 3 h to kill the conidia that adhered to the cells [16, 17]. Our previous study showed that only 1.81% of the viable initial *A. fumigatus* conidia were internalized by A549 cells, and that the infected-cells model could be used to evaluate the intracellular activity of voriconazole against *A. fumigatus* [17].

#### Biomarker of A549 cells and *A. fumigatus*

The viability of infected A549 cells in the presence of voriconazole was evaluated by measuring the cytosolic enzyme lactate dehydrogenase (LDH) released into the culture medium after 24 h of incubation using an LDH cytotoxicity assay kit (Beyotime Institute of Biotechnology, Haimen, Jiangsu, China). The growth of *A. fumigatus* was quantified by collecting culture supernatants and measuring the levels of galactomannan using the Platelia^®^ Aspergillus assay (Bio-Rad, Marnes la Coquette, France) according to the manufacturer’s instructions.

#### Cellular pharmacodynamics of voriconazole

The intracellular activity was examined for extracellular voriconazole concentrations (C_e_) ranging from 0.0155 to 64 mg/L, which is similar to the range of voriconazole ELF concentrations measured in transplant patients. After 24 h of incubation, the A549 cells were lysed with 1 mL of 0.5% Triton X-100, and the released conidia were diluted and then plated onto SDA to count the colony-forming units (cfu). The number of viable *A. fumigatus* conidia in each lysate sample was determined in duplicate incubations on SDA and using the standard plate-count method. The change in the number of cfu from that in the initial intracellular inoculum (at 0 h) was taken as the response to voriconazole and plotted as a function of the extracellular concentration (in logarithmic units). The measured data were fitted with a sigmoid model having a slope of − 1. The curve fittings were performed using GraphPad Prism software (version 5.0, GraphPad Software, San Diego, CA, USA). This sigmoid model allowed us to calculate the static concentration (C_s_) corresponding to the extracellular concentration of voriconazole causing no apparent changes in cfu relative to the initial intracellular inoculum.

### Inhibitory effect sigmoid E_max_ model

An inhibitory effect sigmoid E_max_ model (formula 1) was used to construct the relationship between C_e_ divided by MIC (the C_e_/MIC ratio) and the intracellular antifungal effect at 24 h:

1 $${\text{E~ = ~E}}_{{{\text{min~~}}}} {-}{\text{~}}\frac{{{\text{(E}}_{{{\text{min}}}} ~{-}{\text{~E}}_{{{\text{max}}}} {\text{)~}} \times {\text{~(C}}_{e} {\text{/MIC)}}^{{\text{H}}} }}{{{\text{E}}_{{{\text{50}}}} ^{{\text{H}}} {\text{~ + ~}}({\text{C}}_{e} {\text{/MIC)}}^{{\text{H}}} }}$$

where E is the intracellular antifungal effect of voriconazole, expressed as a proportion of the initial intracellular inoculum, E_min_ is the effect for an infinitely low C_e_/MIC value (this value is > 100%, and corresponds to the increase in cfu compared to the initial intracellular inoculum for an infinity low value of C_e_/MIC). E_max_ is the maximum effect for an infinitely large C_e_/MIC value (this value is < 100% and corresponds to the decrease in cfu compared to the initial intracellular inoculum for an infinitely high value of C_e_/MIC), E_50_ is the extracellular concentration of voriconazole at which E is halfway between E_min_ and E_max_, and H is the slope function. The intracellular pharmacodynamics data were modeled using WinNonlin software (version 4.1, Pharsight, Mountain View, CA, USA).

### Monte Carlo simulation

To further explore the therapeutic implications of these experimental results, Monte Carlo simulation was used to evaluate the probability of different oral dosage regimens achieving the target value of C_e_/MIC to design the appropriate prophylactic regimen of voriconazole in immunosuppressed patients. The MCS was performed by combining experimental results for the infected A549 cells with the data from a previous population pharmacokinetics study. The Monte Carlo simulation was based on

2 $$\frac{{{\text{C}}_{{\text{e}}} }}{{{\text{MIC}}}}{\text{~ = ~}}\frac{{{\text{C}}_{{{\text{plasma}}}} {\text{~}} \times {\text{~R}}_{{{\text{ELF/plasma}}}} }}{{{\text{MIC}}}}$$

where the C_e_/MIC break point of voriconazole comes from the inhibitory effect sigmoid E_max_ model; R_ELF/plasma_ is the ELF-to-plasma ratio of voriconazole concentration, and Voriconazole can penetrate well into the pulmonary ELF in patients, with a R_ELF/plasma_ of 11.5 ± 6.9 (mean ± SD) from published literatures [13, 14]; Voriconazole plasma concentrations and the variability of different dosage regimens in patients were simulated based on the previous population pharmacokinetics model using NONMEM software and twice daily oral dosage regimens of 100, 200, and 300 mg (under the maintenance dose) were simulated [18, 19]. The voriconazole ELF concentration was then calculated as the simulated voriconazole plasma concentration multiplied by R_ELF/plasma_; the MIC distribution of *A. fumigatus* was obtained from the EUCAST (European Committee on Antimicrobial Susceptibility Testing) website.

The results of the Monte Carlo simulation were expressed as the probability of target attainment and the cumulative fraction of response [20]. A cumulative fraction of response value of ≥ 90% was considered to be an appropriate prophylactic regimen [21].

### Statistical analysis

In the present study, numbers of cfu were converted to logarithms, base 10, for statistical analysis. All experimental values were expressed as the mean ± standard deviations of three independent experiment.

## Results

### Minimum inhibitory concentrations

The voriconazole MICs for *A*. *fumigatus* strains AF293 and AF26 were 0.19 and 0.064 mg/L, respectively.

### Biomarker

**Fig. 1:**
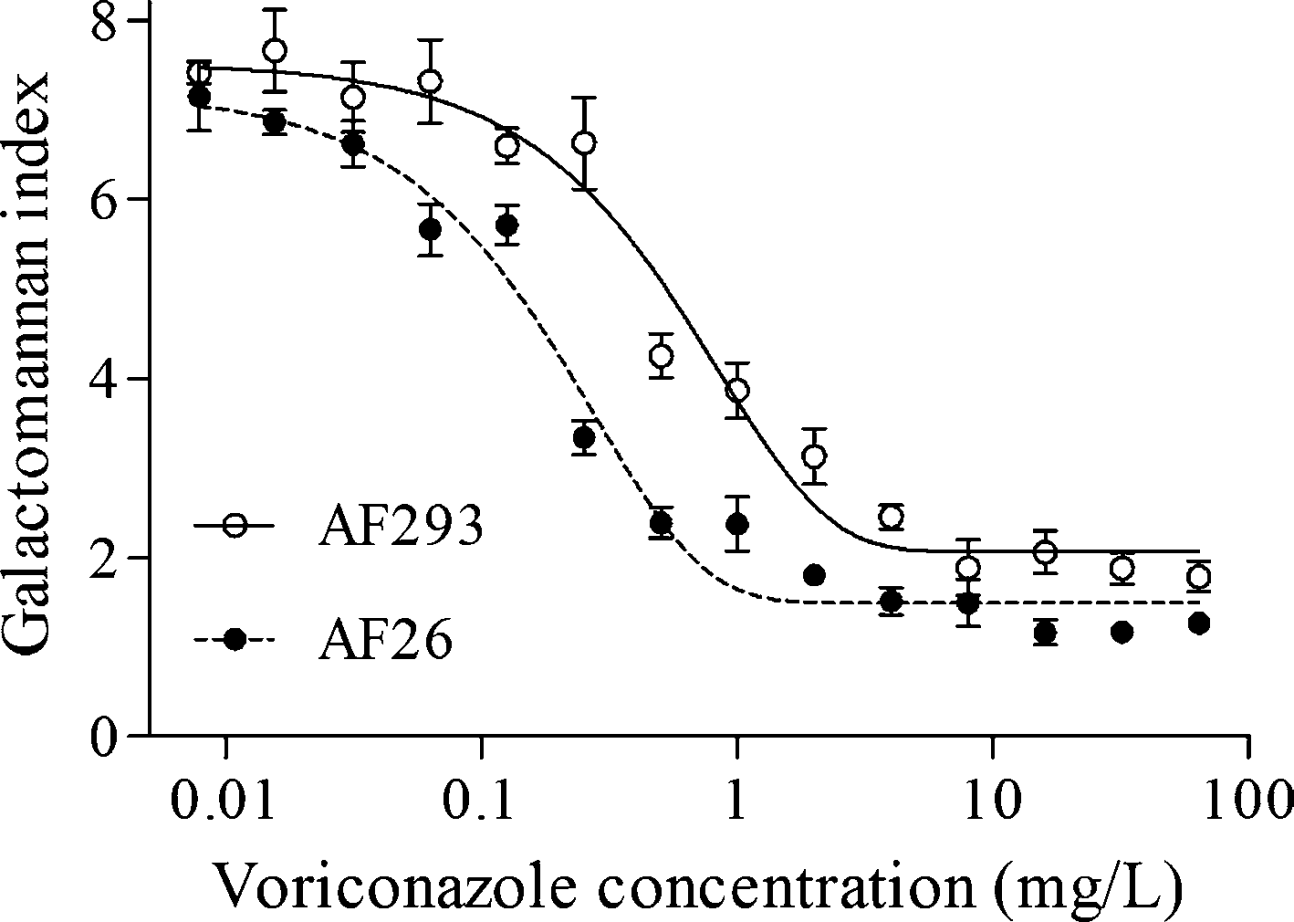
Concentration—galactomannan index relationships for voriconazole against two strains of *A. fumigatus* with differing MIC values in infected A549 model. The solid and dashed lines were fitted by the sigmoid function

We examined the viability of infected A549 cells for C_e_ values ranging from 0.0155 to 64 mg/L by measuring the LDH released into the medium. The amount released remained similar to the control values and lower than 11.5% over the whole range of concentrations investigated, which was therefore these levels considered when performing further experiments. Additionally, we found that the kinetics profile of galactomannan was correlated with C_e_ and that voriconazole induced a concentration-dependent decrease in the galactomannan index for both AF293 and AF26 (Fig. 1).

### Cellular pharmacodynamics of voriconazole against AF293 and AF26

**Fig. 2:**
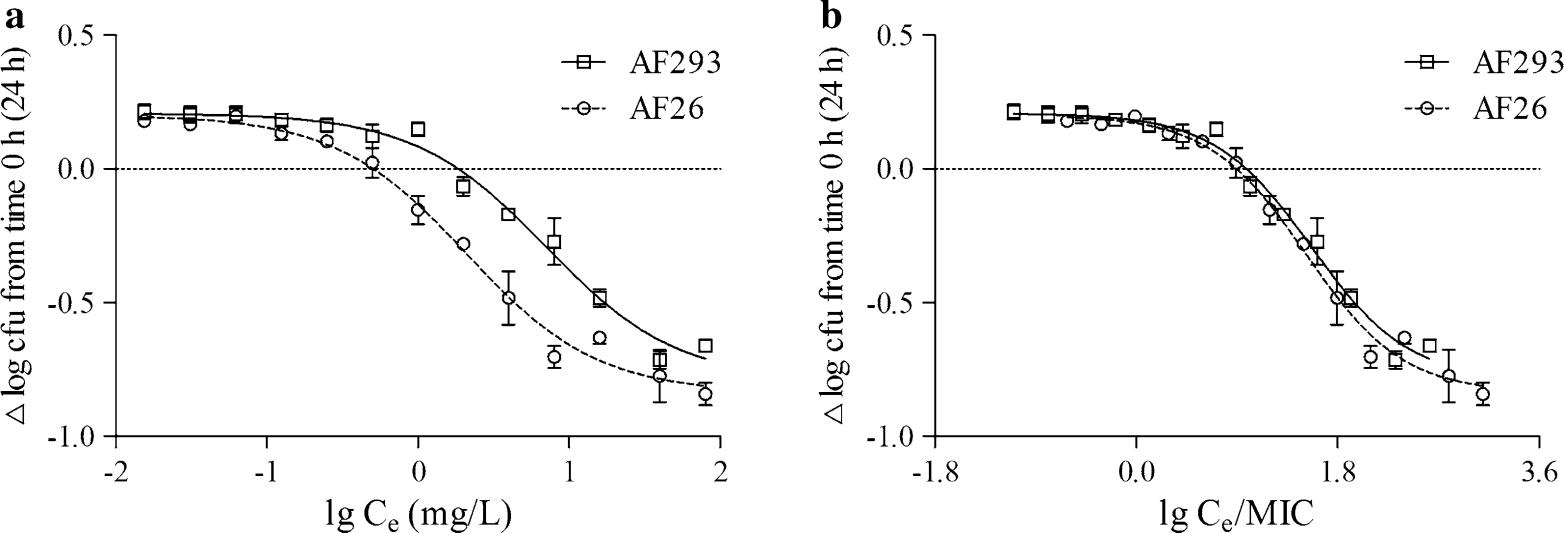
Goodness of fit fitted by sigmoidal function: extracellular concentration-dependent activity of voriconazole against intracellular *A. fumigatus*. The ordinate shows the change in the value of cfu (∆lg cfu) per mL of cell lysate after 24 h incubation compare with the initial intracellular conidia. **a** Data were plotted against the extracellular concentration (mg/L) of voriconazole. **b** Data were plotted against the ratio of extracellular concentration divide MIC value

The initial intracellular inoculum (at 0 h) was 1–2 × 10^5^ cfu/mL per plate for the two strains investigated in this study. The voriconazole activity developed in a concentration-dependent manner, with a sigmoid function providing a satisfactory fit to the data for the two strains (Fig. 2a). The E_min_ values were similar for the two strains (approximate increases of 0.2 lg cfu), and reduced the intracellular inoculum by 0.79–0.84 lg cfu compared to the initial value at a high C_e_ (E_max_). The number of intracellular *A. fumigatus* conidia was unchanged for the two strains compared to the initial intracellular inoculum at a C_s_ of 1.86 mg/L for AF293 and 0.52 mg/L for AF26, and E_50_ was 7.05 mg/L for AF293 and 2.11 mg/L for AF26 (Table 1). A particularly interestingly observation was that when C_e_ was expressed in multiples of the corresponding MIC (i.e., C_e_/MIC ratio), the concentration–response curves of voriconazole against AF293 and AF26 became extraordinary close on the coordinate axis, which demonstrates the importance of MIC values to intracellular activity of voriconazole (Fig. 2b).

**Table 1:** The regression parameters and statistical analysis of the extracellular concentrations-activity curves illustrated in Fig. 2

Strain	E_max_ (95%CI)^a^	E_min_ (95%CI)^b^	EC_50_ (95%CI)^c^	C_s_^d^	R^2e^
AF293	− 0.79 (− 0.86 ~ − 0.72) lg cfu	0.21 (0.18 ~ 0.24) lg cfu	7.05 mg/L (5.36 ~ 9.27) 36.71 × MIC (27.91 ~ 48.30)	1.86 mg/L (9.69 × MIC)	0.9813
AF26	− 0.84 (− 0.89 ~ − 0.78) lg cfu	0.20 (0.16 ~ 0.24) lg cfu	2.11 mg/L (1.63 ~ 2.73) 32.93 × MIC (25.39 ~ 42.70)	0.52 mg/L (8.32 × MIC)	0.9828

^a^cfu decrease (in lg units) at 24 h from the corresponding original cellular conidia, as extrapolated for infinitely high concentrations of voriconazole (note that a larger maximal relative activity corresponds to a more negative value of E_max_)

^b^cfu increase (in lg units) at 24 h from the corresponding original cellular conidia, as extrapolated for infinitely low concentrations of voriconazole

^c^Exposure (in extracellular concentration and multiple of corresponding MIC) causing a reduction halfway between the E_min_ and E_max_ values, as obtained from the hill equation by using a slope factor of − 1

^d^Exposure (in extracellular concentration and multiple of corresponding MIC) resulting in no apparent pathogen growth (the number of cfu was identical to that of the original cellular conidia), as determined by graphical interpolation

^e^R is coefficient of correlation

### Cellular PK/PD of voriconazole

**Fig. 3:**
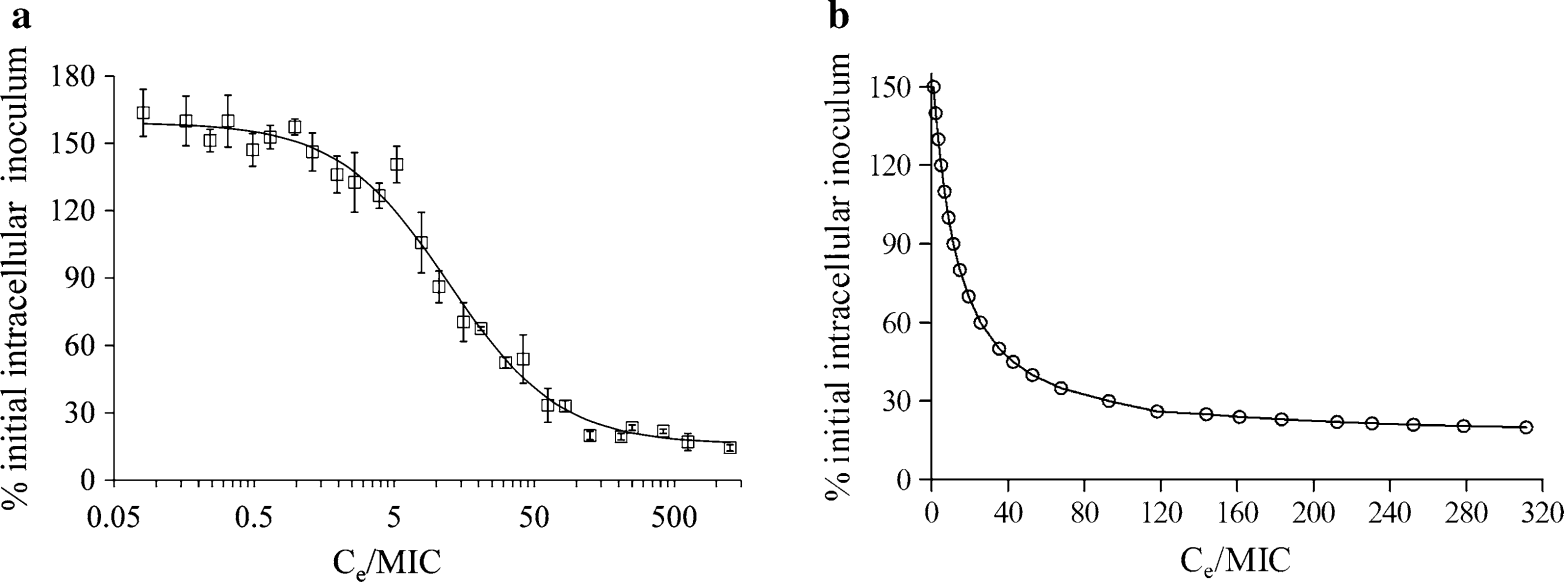
Relationship between the total voriconazole C_e_/MIC and the intracellular antifungal effect as measured by the proportion of initial incubation. **a** The solid black line is the fit of an mathematical model to data from the infected A549 model, and the calculated equation by WinNonlin software is: effect index = 159.4 − 143.4 × (C_e_/MIC)^1.1^/(12.2^1.1^ + (C_e_/MIC)^1.1^), R^2^ = 0.9738. **b** The relationship between C_e_/MIC breakpoint and intracellular antifungal effect

All of the data could be fitted well by the inhibitory effect sigmoid E_max_ model, with a correlation coefficient of 0.97 and a slope of 1.10 (Fig. 3a). The model showed that progressively higher C_e_/MIC values resulted in a progressive decrease in the number of *A. fumigatus* conidia in cells. Compared to the initial intracellular inoculum, the number of intracellular *A. fumigatus* conidia could be suppressed by 84.01% or increased by 59.36%, respectively, within 24 h, corresponding to the asymptotic maximum and minimum C_e_/MIC values. C_e_/MIC values of 8.93, 14.9, 35.53, and 311.30 resulted in 100, 80, 50, and 20% of the initial intracellular number of *A.* *fumigatus* conidia surviving in the A549 cells (Fig. 3b).

### Monte Carlo simulation results

Monte Carlo simulation was used to evaluate the probability of different dosage regimens achieving the target value of C_e_/MIC = 35.53. Table 2 lists the probability of target attainments for specific MIC values ranging from 0.0625 to 16 mg/L for each dosage regimen. For the MIC of 0.125 mg/L, the values of probability of target attainments for voriconazole dosage regimens of 100, 200, and 300 mg administered twice daily via the oral route were 85.27, 92.94 and 98.03%, respectively. For *A.* *fumigatus*, voriconazole oral dosages of 100, 200, and 300 mg twice daily yielded estimated cumulative fraction of response values of 85.85, 91.48 and 96.19%, respectively (Table 2).

**Table 2:** The probability of target attainment values at a certain minimum inhibitory concentration and the cumulative fraction of response values for *A. fumigatus* at different dosage regimens

Dosage regimens	Probability of target attainment (%) at different minimum inhibitory concentrations										CFR (%)
	0.03125 mg/L	0.0625 mg/L	0.125 mg/L	0.25 mg/L	0.5 mg/L	1 mg/L	2 mg/L	4 mg/L	8 mg/L	16 mg/L	
100 mg/12 h, p.o.	99.25	96.35	85.27	63.9	36.39	14.92	4.14	0.72	0.099	0.006	84.45
200 mg/12 h, p.o.	99.65	98.21	92.94	80.01	58.78	34.15	15.28	4.92	1.15	0.21	91.48
300 mg/12 h, p.o.	99.98	99.67	98.03	92.25	77.55	54.15	29.07	11.35	3.07	0.61	96.19

*CFR* cumulative fraction of response

## Discussion

Pulmonary epithelial cells invaded by *A. fumigatus* conidia in human body is the first step of causing IPA, and IPA is still related to high rates of morbidity and mortality after the utilization of antifungal agents [3–5]. Therefore, how to design the optimum prophylactic dosage regimen to prevent IPA is an intractable problem in clinical practice. Voriconazole has a strong activity against and it is vital to explore the intracellular activity of voriconazole against *A. fumigatus* in pulmonary epithelial cells. This study built the cells model infected by *A. fumigatus* to investigate, firstly, the PK/PD property of voriconazole in infected cells, and then use the Monte Carlo simulation and PK/PD breakpoint to design the prophylactic dosage regimen. This research provided a potential mechanism of voriconazole preventing IPA on a cellular level and provided a novel theoretical and experimental method for the designation of voriconazole prophylactic dosage regimen and the reduction of IPA morbidity.

Previous pharmacodynamics studies have used galactomannan (a major constituent of aspergillus cell walls) [22] as a biomarker for assessing the antifungal activity against *A.* *fumigatus* [23–25]. The present study found a distinct relationship between the voriconazole concentration and the galactomannan index for the two *A. fumigatus* strains (Fig. 1). In contrast to previous studies, [23–25] we did not calculate the pharmacodynamics parameters based on the relationship between the voriconazole concentration and the galactomannan index. One of the limitations in semiquantitative assessment is that the content of galactomannan in the medium cannot precisely reflect the number of *A. fumigatus* conidia in cells. Thus, the number of *A. fumigatus* conidia in A549 cells was determined in this study by performing quantitative culturing in a subsequent step.

*A. fumigatus* conidia in A549 cells were exposed for 24 h to voriconazole over a wide range of concentrations to determine the overall pattern of intracellular activity. The data obtained in this study of voriconazole cellular pharmacodynamics properties have not been reported previously in detail. The E_max_ values of voriconazole for the AF293 and AF26 strains were 0.79 and 0.84 lg cfu, respectively, showing a decrease compared with the initial intracellular inoculum (Table 1), which meant that 78.20 and 85.57% of intracellular *A. fumigatus* conidia were suppressed within 24 h for AF293 and AF26, respectively. At the infinitely low C_e_, the number of intracellular *A. fumigatus* conidia increased by 63.63 and 51.28% for AF293 and AF26, respectively. When the data were plotted against C_e_, the C_s_ calculated using the sigmoid model was 0.52 mg/L for AF26 and 1.86 mg/L for AF293, respectively. A particularly interesting observation was how close the intracellular pharmacodynamics parameters of voriconazole against AF293 and AF26 became when the data were plotted against C_e_/MIC, with C_s_ was 8.32-fold MIC for AF26 and 9.69-fold MIC for AF293, and E_50_ was 32.93-fold MIC for AF26 and 36.71-fold MIC for AF293 (Table 1 and Fig. 2b). This result suggests that C_e_ and the inherent susceptibility of *A. fumigatus* were the key factors influencing the voriconazole activity against *A. fumigatus* in the intracellular milieu. In other words, the C_e_/MIC ratio might be a crucial determinant of the voriconazole intracellular activity against *A.* *fumigatus.*

Through the above analysis we were able to demonstrate that C_e_/MIC might be the best predictor of the voriconazole intracellular antifungal effect. To our knowledge, the present study is the first to use infected A549 cells combined with an inhibitory effect sigmoid E_max_ model to study the PK/PD breakpoint. All of the data for the voriconazole intracellular activity against AF293 and AF26 are summarized in Fig. 3. The model showed that up to 84.01% of the initial intracellular inoculum was suppressed by voriconazole within 24 h (Fig. 3a). Given that 15.99% of the initial intracellular inoculum (about 4.4 lg cfu conidia) survived in the cells indicates that voriconazole could not completely suppress intracellular *A. fumigatus* even at higher incubation concentrations. This phenomenon may be due to the saturation of voriconazole absorption and the low voriconazole concentration in A549 cells [17, 26].

In the present study, we firstly found that the PK/PD breakpoint in the infected A549 model predicting the intracellular antifungal effect of voriconazole was C_e_/MIC = 35.53, which was associated with a 50% suppression of intracellular *A.* *fumigatus* (Fig. 3b). Previous studies have shown that PK/PD parameters of antimicrobial agents could be useful for designing dosage regimens. Thus, C_e_/MIC = 35.53 was utilized as the PK/PD index for the subsequent Monte Carlo simulation to design the prophylactic regimen for voriconazole. For cases of IPA that are mostly caused by airborne *A. fumigatus*, knowledge of the epidemiology of *A. fumigatus* is pivotal and could be used to underpin prophylactic strategies. The value of cumulative fraction of response is an estimate of the proportion of a population achieving the target PK/PD index. The results of Monte Carlo simulation showed that the effective suppression of intracellular *A. fumigatus* at least requires an oral dosage of 200 mg of voriconazole twice daily, which yielded a CFR value of 91.48% (Table 2). Actually, the Monte Carlo simulation result has been largely confirmed in clinical applications, because the dosage regimen has been widely demonstrated to be effective at preventing IPA [27–31]. For example, an oral dosage of 200 mg of voriconazole twice daily has been described as an appropriate and effective prophylactic agent in children and adults with acute myeloid leukemia or myelodysplastic syndrome and in those undergoing hematopoietic stem cell transplantation or solid-organ transplantation [32–35]. This study has also been demonstrated that the C_e_/MIC breakpoint is a rational metric to use when designing dosage regimens for patients receiving voriconazole for prophylaxis.

The pulmonary ELF is on the surface of pulmonary epithelial cells and the concentration of an antifungal agent within the ELF is critical to the prophylaxis and treatment of the early stages of IPA [12, 36]. Clinically, patients typically take 200 mg of voriconazole orally twice daily—which is the recommended dosage regimen on the package insert—during prophylaxis applications. However, there was a lot of sub-therapeutic concentrations among some patients taking the oral dosage of 200 mg twice daily. For example, the concentration was adequate (< 1.0 mg/L) in 45% of allogeneic hematopoietic stem cell transplantation recipients [37]. Based on in vitro susceptibility data, the MIC_90_ (MIC at which 90% of the strain was inhibited) for voriconazole against *A. fumigatus* was 0.5 mg/L [38] and the C_e_/MIC value was 35.53. We can thus calculate that the target voriconazole concentration in ELF for prophylaxis is 17.77 mg/L. However, the actual voriconazole concentration in ELF is difficult to measure, and it is much easier to measure it in plasma. According to the mean value of R_ELF/plasma_, we calculated that the target voriconazole plasma concentration was 1.55 mg/L. A prospective, observational study showed that voriconazole trough concentration higher than 1.5 mg/L was the most effective for prophylaxis [39], which was close to the result of present study. Notably, when applying these results to clinical practice, we should characterize the local epidemiology of *A. fumigatus* and monitor the concentration of voriconazole in ELF or plasma, which would ensure the C_e_/MIC > 35.53 for voriconazole against most isolates of *A.* *fumigatus* in patients receiving voriconazole prophylaxis. However, the relationship between C_e_/MIC and clinical efficacy still needs to be verified in further clinical studies.

This study was subject to some major limitations. (1) The R_ELF/plasma_ value from the previous two studies may be somewhat unreliable due to the inclusion of only 20 samples; (2) currently the mechanism underlying how voriconazole is loaded into pulmonary epithelial cells remains unknown. The present study used extracellular voriconazole concentrations modelled on those reported in ELF fluid, whereas intracellular voriconazole may come from both ELF and plasma; (3) for the reliability of the results, additional experiments should be carried out in pulmonary epithelial cells different from A549. However, the method for building a cell model infected by *A.* *fumigatus* in this study is based on the study of Wasylnka et al. [16]., and the this model was proved to be reasonable and reliable in previous studies [17, 26, 40, 41]. Therefore, this cells model could afford a reliable result for this study.

## Conclusions

Voriconazole exhibits concentration-dependent activity against intracellular *A.* *fumigatus*, and this study found that up to 84.01% of the intracellular inoculum was suppressed compared with the initial intracellular inoculum, and 50% suppression of the initial intracellular *A.* *fumigatus* was observed at a C_e_/MIC breakpoint of 35.53. Additionally, this study identified the target voriconazole concentration in ELF and plasma was 17.77 and 1.55 mg/L, respectively, in patients receiving voriconazole prophylaxis. The present study has revealed the potential mechanism of voriconazole prophylaxis from the perspective of cellular PK/PD model, as well as provided a method for designing or optimizing prophylactic dosage regimens for voriconazole in high-risk immunosuppressed patients based on Monte Carlo simulation and the target voriconazole concentration. The reported findings have significant implications for reducing the morbidity of IPA in immunosuppressed patients.

## Abbreviations

IPA: Invasive pulmonary aspergillosis
ELF: Epithelial lining fluid
A549: Human pulmonary epithelial cells
MIC: Minimum inhibitory concentration
LDH: lactate dehydrogenase
cfu: Colony forming units
C_e_/MIC: The ratio of extracellular voriconazole concentration divided minimum inhibitory concentration
PK/PD: Pharmacokinetic/pharmacodynamics
SDA: Sabouraud dextrose agar
C_s_: Static concentration
